# Effects of antenatal education on maternal anxiety and depression in pregnancy and postpartum period in Italy: modest and transient symptom reductions

**DOI:** 10.3389/fpsyg.2025.1724202

**Published:** 2026-02-12

**Authors:** Laura Camoni, Fiorino Mirabella, Antonella Gigantesco, Gemma Calamandrei, Alberto Stefana, Perinatal Mental Health Network group, Franca Aceti, Ilaria Adulti, Pietro Bagolan, Gina Barbano, Antonello Bellomo, Marina Cattaneo, Elda Cengia, Flavia Adalgisa Distefano, Angela Fabiano, Alice Fent, Nicoletta Giacchetti, Laura Giusti, Antonella Grillo, Teresa Grimaldi, Loredana Messina, Marianna Mazza, Angelo Marcheggiani, Cinzia Niolu, Giovanna Picciano, Maria Pistillo, Rossana Riolo, Rita Roncone, Gabriele Sani, Melania Severo, Martina Smorti, Damiana Tomasello

**Affiliations:** Osservatorio Multicentrico per la Depressione Perinatale, Servizio di psicopatologia perinatale, Sapienza Università di Roma, Policlinico Umberto I, Roma, Italy; Osservatorio Multicentrico per la Depressione Perinatale, Università di Tor Vergata, Roma, Italy; Ospedale Pediatrico Bambino Gesù, Unità Operativa di Psicologia Clinica e diagnosi prenatale, Roma, Italy; UOC Infanzia Adolescenza Famiglia e Consultori, Distretto Treviso nord-sede di Oderzo, Azienda ULSS 2 Marca trevigiana, Treviso, Italy; Osservatorio Multicentrico Depressione Perinatale, UOC Psichiatria, Università di Foggia, Foggia, Italy; Consultorio familiare di Treviglio, ASST Bergamo Ovest, Bergamo, Italy; UOC Ostetricia e Ginecologia Ospedale di Belluno Azienda ULSS 1, Belluno, Italy; UOC di Ginecologia ed Ostetricia dell'Ospedale Cristo Re, Roma, Italy; Divisione Materno Infantile dell'Azienda Ospedaliera di Rilievo Nazionale e di Alta Specializzazione (ARNAS) “Garibaldi”, Catania, Italy; UOC Ostetricia e Ginecologia Ospedale di Belluno Azienda ULSS 1, Belluno, Italy; Servizio di psicopatologia perinatale Sapienza Università di Roma, Policlinico Umberto I, Osservatorio Multicentrico per la Depressione Perinatale, Roma, Italy; Università degli Studi de L'Aquila, Dipartimento di Medicina Clinica, Sanità Pubblica, Scienze della Vita e dell'Ambiente - MeSVA, L'Aquila, Italy; Divisione Materno Infantile - ASP Catania, Catania, Italy; Ospedale Pediatrico Bambino Gesù, Unità Operativa di Psicologia Clinica e diagnosi prenatale, Roma, Italy; UOC Ostetricia e Ginecologia dell'Ospedale Buccheri La Ferla Fatebenefratelli, Palermo, Italy; Osservatorio Multicentrico Depressione Perinatale, UOC Psichiatria, Policlinico A. Gemelli, Roma, Italy; Consultorio di Campobasso - Ambulatorio di Ostetricia e Ginecologia, Campobasso, Italy; Osservatorio Multicentrico per la depressione perinatale, Università di Tor Vergata, Roma, Italy; Consultorio di Campobasso - Ambulatorio di Ostetricia e Ginecologia, Campobasso, Italy; UOC Ostetricia e Ginecologia, ASP Enna, Enna, Italy; Ambulatorio genitori senza depressione, ALSS 8-Berica, Vicenza, Italy; Università degli Studi de L'Aquila, Dipartimento di Medicina Clinica, Sanità Pubblica, Scienze della Vita e dell'Ambiente - MeSVA, L'Aquila, Italy; Osservatorio Multicentrico Depressione Perinatale, UOC Psichiatria, Policlinico A. Gemelli, Roma, Italy; Osservatorio Multicentrico Depressione Perinatale, UOC Psichiatria, Università di Foggia, Foggia, Italy; Dipartimento di Patologia Chirurgica, Medica, Molecolare e dell'Area Critica, Università di Pisa, Pisa, Italy; Divisione Materno Infantile - ASP Catania, Catania, Italy; Center for Behavioural Sciences and Mental Health, National Institute of Health, Rome, Italy

**Keywords:** anxiety, childbirth classes, depression, expectant parent classes, prenatal education

## Abstract

**Introduction:**

Antenatal classes have increasingly been integrated into healthcare practices in most middle- and high-income countries over recent decades. The aim of the present study was to compare levels of anxiety and depressive symptoms during pregnancy and the postpartum period among (a) women who attended antenatal classes and (b) women who did not participate in antenatal education.

**Methods:**

We analyzed 9,689 perinatal respondents recruited in eight Italian regions between October 2021 and December 2024. Each participant was assessed once, during their pregnancy (*n* = 4,169) or their postpartum period (*n* = 5,520), and completed the General Anxiety Disorder-7 (GAD-7) and Edinburgh Postnatal Depression Scale (EPDS) scales. The cut-off scores to identify women at risk for depression and anxiety were ≥12 and ≥10 for EPDS and GAD-7, respectively. Propensity scores based on socio-demographic and clinical covariates were estimated with multiple imputations and inverse-probability-of-treatment weighting (IPTW).

**Results:**

Attendance was frequent (47%). Crude models showed that, during pregnancy, class participants had lower mean scores (ΔGAD = −0.8; ΔEPDS = −1.0) and markedly lower odds of screening positive (OR = 0.58 for anxiety; 0.45 for depression). After IPTW adjustment these associations weakened and became non-significant (pregnancy OR = 0.86, 95% CI 0.54–1.35 for anxiety; 0.64, 0.38–1.10 for depression); all post-partum IPTW estimates were similarly null (ORs 0.96 and 0.83, CIs span 1). E-values (1.9–2.5) indicated that moderate unmeasured confounding could erase the residual pregnancy effects.

**Conclusions:**

Our results suggest antenatal education classes are modestly effective in reducing anxiety and depressive symptoms during pregnancy. However, these modest prenatal improvements attenuate after adjustment and do not persist into the postpartum period. This indicates a need for standardized, evidence-based antenatal education that is integrated into broader psychosocial support frameworks.

## Introduction

Pregnancy and childbirth are characterized by complex hormonal, physical, emotional, and psychological changes in women's lives (Jee and Sawal, 2024; Olza et al., 2018). During pregnancy, expectant mothers often experience anxiety, uncertainty, and fear regarding pregnancy, labor, delivery, and their ability to care for a newborn (Sheen and Slade, 2018; Wilska et al., 2021). Therefore, it is unsurprising that nearly one in four pregnant women experiences symptoms or disorders related to anxiety (Fawcett et al., 2019) or depression (Yin et al., 2021). These conditions frequently co-occur (Ou et al., 2025; Shen et al., 2024) and often increase in prevalence during the postpartum period (Astbury et al., 2025). Antenatal anxiety and mood disorders have been associated with adverse obstetric and neonatal outcomes (Runkle et al., 2023), maternal mortality, suicide, self-harm (Howard and Khalifeh, 2020), problematic mother-infant bonding (O'Dea et al., 2023), and psychiatric symptoms in offspring (Chen et al., 2024). Collectively, these findings underscore the importance of early screening and assessment (Weingarten and Osborne, 2024), as well as preventive interventions (Motrico et al., 2023; Wu et al., 2025).

Regarding preventive nonclinical interventions, antenatal education (also known as antenatal classes, expectant parent classes, antenatal parenthood education, prenatal classes, childbirth preparation, or childbirth classes) has increasingly been integrated into healthcare practices in most middle- and high-income countries over recent decades (Downer et al., 2020). The WHO (2018) recommends several context-specific interventions, including (i) childbirth education workshops, covering topics such as childbirth-related fear and pain, pharmacological pain-relief options and their effects, nonpharmacological pain-management strategies, benefits and risks of cesarean vs. vaginal delivery, and indications and contraindications for cesarean sections; (ii) nurse-led applied relaxation programs, including group discussions about pregnancy-related anxiety and stress, the rationale for applied relaxation, and training in deep-breathing and other relaxation techniques; (iii) psychosocial couple-based prevention programs, focusing on emotional self-regulation, conflict resolution, problem-solving, communication skills, and mutual support strategies to promote positive co-parenting; and (iv) psychoeducation sessions led by therapists and midwives that provide information about fear and anxiety, pain and childbirth fears, normalization of individual responses, stages of labor, hospital procedures, the birth process, and pain-relief options.

Although antenatal education programs vary nationally and internationally in content and delivery methods, they typically serve the dual purpose of familiarizing women and their partners with essential aspects of pregnancy and childbirth and preparing them for newborn care [National Institute for Health and Care Excellence (NICE), 2021; WHO, 2016]. The importance of antenatal education in enhancing understanding and supporting women's transition and adaptation to motherhood has also been highlighted in a recent umbrella review and framework (Matvienko-Sikar et al., 2025). Additionally, the need for standardization of these educational programs, while allowing room for personalization to avoid scenarios lacking evidence-based recommendations, has been emphasized (AlKhunaizi et al., 2025; Ferri et al., 2024).

Regarding the effectiveness of antenatal education, meta-analytic findings demonstrate that these programs improve childbirth self-efficacy (Demirci et al., 2023; Zaman et al., 2025; Zanetti et al., 2024), decrease fear of childbirth (Alizadeh-Dibazari et al., 2023; Zaman et al., 2025), reduce rates of planned cesarean sections (Hooper et al., 2025; Zaman et al., 2025; Zanetti et al., 2024), and decrease pain intensity during labor (Alizadeh-Dibazari et al., 2023). Regarding mental health outcomes, particularly anxiety and depression, insufficient evidence exists due to the small number of studies, high heterogeneity in interventions and outcomes, and elevated risk of bias (Alizadeh-Dibazari et al., 2023; Brixval et al., 2015; Hong et al., 2021). Nevertheless, recent non-meta-analytic reviews suggest antenatal education effectively reduces anxiety levels during pregnancy, especially in primigravidas (Athinaidou et al., 2024; Rahmawati et al., 2025). Evidence from psychoeducational prenatal classes indicates reductions in anxiety and improvements in self-efficacy, supporting a plausible pathway for perinatal adjustment (Diotaiuti et al., 2022).

The present study aimed to compare levels of anxiety and depressive symptoms during pregnancy and the postpartum period among (a) women who attended antenatal classes and (b) women who did not participate in antenatal education, within a large perinatal sample. Analyses controlled for maternal age, education, economic status, employment, marital status, past psychiatric diagnosis, current psychotropic medication use, perceived social support, pregnancy trimester, pregnancy planning, assisted conception, parity, and survey period.

## Methods

### Procedures

This ongoing study (initiated in 2021) is organized by the Center for Behavioral Sciences and Mental Health at the Istituto Superiore di Sanità (Italian National Institute of Health; ISS). In September 2021, the ISS launched the Italian Network for Perinatal Mental Health to track anxiety and depression throughout the perinatal period, and to examine associated risk and protective factors. The Network comprises 16 sites (including obstetrics and gynecology units, psychiatric hospitals, and maternal–child health centers) across eight Italian regions. Recruitment occurred voluntarily during gynecological or obstetric routine appointments. Each participant received a full explanation of the study's aims and procedures and provided written informed consent, with the right to withdraw at any time without affecting their medical care. Each participant was assessed once, at any time during their pregnancy (*n* = 4,169 pregnant women) or their postpartum period (*n* = 5,520 new mothers). The protocol received ethical approval from the Ethics Committee of the ISS (Protocol No. 0024542, 21 June 2021) and adhered to the principles stated in the “Declaration of Helsinki.” The data analyzed in this article were collected between October 2021 and December 2024.

### Antenatal education classes

Antenatal education classes are programs designed for expectant couples, led by professionals such as midwives, psychologists, gynecologists, and pediatricians. They generally aim to provide practical and psychological tools to help women navigate pregnancy, childbirth, and the postpartum period with greater awareness. In Italy, the sessions typically address topics related to a woman's physical and mental wellbeing, nutrition, the stages of labor, and pain management techniques, both pharmacological and natural, with particular attention to the partner's role. Newborn care, breastfeeding, and adjusting to a new family are also addressed, with practical exercises such as breathing, relaxation, and baby massage. The courses are held in small groups to encourage sharing experiences and typically consist of 5–10 sessions lasting between an hour and a half and 2 h, typically beginning between the twenty-third and thirty-second week of pregnancy. Participants in the present study attended prenatal courses organized by various institutions; although these courses followed general guidelines for antenatal education, differences in structure and content may have existed, for which detailed data were not available. We also lacked data on the number of days/weeks between the final class and the assessment.

### Measures

A structured patient questionnaire (Mirabella et al., 2016) was used to collect sociodemographic, clinical, and pregnancy-related data (e.g., age, marital status, psychiatric history, and perceived social support, recourse to medically assisted procreation). The instrument has been employed in previous perinatal studies (e.g., Cena et al., 2020, 2021a,b,c, 2022), which consistently found that several of these variables were independently and statistically significantly associated with the severity of anxiety and depressive symptoms, after adjustment for potential confounders.

Depressive symptoms were assessed with the 10-item Edinburgh Postnatal Depression Scale (EPDS; Cox et al., 1987), a self-report measure that evaluates mood and anhedonia symptoms over the preceding 2 weeks. Although two-factor models showed the best fit for the EPDS in both antepartum and postpartum groups (Stefana et al., 2024), the most reliable score variance derives from a general factor for each scale (Stefana et al., 2023). Each item is scored from 0 to 3, resulting in a total score from 0 to 30. A cut-off of ≥12 indicates clinically relevant depressive symptomatology in perinatal populations (Benvenuti et al., 1999; Camoni et al., 2023). In our sample, the EPDS showed strong psychometric performance, with Cronbach's alpha = 0.90 in both pregnancy and postpartum and McDonald's omega total = 0.93 in pregnancy and 0.92 in postpartum. Moreover, in the Italian validation study (Benvenuti et al., 1999), the EPDS demonstrated sensitivity of 0.86 and specificity of 0.78 for identifying probable depression.

Anxiety severity was measured using the 7-item Generalized Anxiety Disorder scale (GAD-7; Spitzer et al., 2006), which assesses the frequency of anxiety-related symptoms over the prior 2 weeks on a 4-point Likert scale (0 = “not at all” to 3 = “nearly every day”). Total scores range from 0 to 21, with higher scores indicating greater anxiety severity; a threshold of ≥10 suggests clinically significant anxiety (Spitzer et al., 2006). In our sample, the GAD-7 showed high internal consistency, with Cronbach's alpha = 0.90 and McDonald's omega total = 0.93 in pregnancy and 0.88 and 0.92, respectively, in postpartum. The Italian version of the GAD-7 demonstrates strong psychometric properties and measurement invariance between antepartum and postpartum samples (Stefana et al., under review).

### Statistical analysis

All analyses were conducted in R 4.5.1 (R Core Team, 2025). We retained 9,689 records with non-missing antenatal-class-attendance status. Twelve prespecified socio-demographic and clinical covariates (Supplementary Table 1) were entered as potential confounders; continuous covariates were mean-centered.

Item-level missingness was < 6%. We applied multivariate imputation by chained equations (m = 10) using predictive mean matching for numeric and logistic regression for binary variables. The imputation model contained all covariates and relevant outcome.

Separate linear regressions predicted EPDS and GAD-7 total scores. Logistic models predicted binary risks (depression: EPDS>12; anxiety: GAD-7 > 8). A multinomial-logit model predicted a four-level symptom profile (None, Depression-only, Anxiety-only, Comorbid). Each model included antenatal-class attendance (Yes vs. No), period, their interaction, and the covariate block. For binary outcomes we report odds ratios (ORs) with 95% CIs; for the multinomial outcome, relative-risk ratios (RRRs). Estimated marginal means were obtained with *emmeans*; risk differences (RDs) and numbers-needed-to-treat (NNT = 1/|RD|) were derived on the probability scale. Model assumptions (linearity, homoscedasticity, and normality or deviance residuals) were verified graphically.

To address self-selection for the binary outcomes (any anxiety, any depression, comorbid), we estimated ATE propensity scores with *WeightIt* using a main-effects logistic regression of attendance on period + the 12 covariates (method = “ps,” estimand = “ATE”). We applied the resulting unstabilized inverse-probability-of-treatment weights in survey-weighted GLMs (*svyglm*, quasi-binomial link) with robust (sandwich) standard errors. No weight stabilization or trimming/truncation was performed. IPTW models were fit in each imputed dataset and pooled with *MIcombine* following Rubin's rules.

Using the same covariate set and ATE IPTW, moderation was tested with survey-weighted linear models including Class × Support/Parity/Education interactions; simple effects were obtained with *emmeans*. Mediation was evaluated with *mediate* using weighted models for the mediator and outcome, with 1,000 non-parametric bootstraps for CIs.

As a sensitivity analysis, E-values (VanderWeele and Ding, 2017) quantified the minimum unmeasured-confounding strength (risk-ratio scale) required to fully explain observed associations.

## Results

### Participants

**Figure 1:**
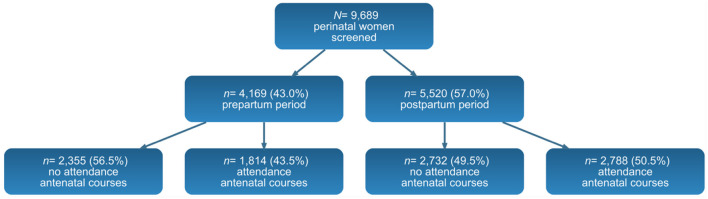
Flow chart from screening to antennal courses analyzed by perinatal period.

The sample comprised *N* = 9,689 perinatal women. Of these, 4,169 (43.0%) were assessed during pregnancy [no attendance *n* = 2,355 [56.5%]; attendance *n* = 1,814 [43.5%]] and 5,520 (57.0%) during the postpartum period [no attendance *n* = 2,732 [49.5]; attendance *n* = 2,788 [50.5%]] (see Figure 1). During pregnancy, antenatal class attenders were significantly more likely to be Italian nationals, hold tertiary education, and be employed, compared with non-attenders. They were less likely to have previous children, report an unplanned pregnancy, or be taking psychotropic medication. Attenders also reported higher social support (all *p* < 0.001). A similar pattern was observed postpartum: class attenders were again more often Italian, tertiary educated, and employed, and less likely to have other living children or report unplanned pregnancies. They showed higher partner and family support and lower psychotropic medication use (*p* < 0.05). Participant characteristics are detailed in Supplementary Tables 1, 2.

### Prevalence of Antenatal-Class Attendance

Attendance was reported by 43.5% of women who were still pregnant (*n* = 1,814) and 50.5% of those surveyed after delivery (*n* = 2,788).

### Primary outcomes: symptom severity and binary screening

**Figure 2:**
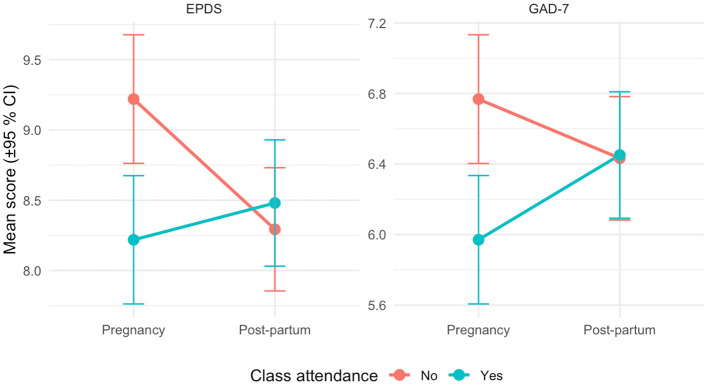
Predicted EPDS and GAD-7 mean symptom scores (±95% CIs) by antenatal-class attendance and period.

For anxiety, women who attended antenatal classes during pregnancy recorded GAD−7 scores that were, on average, 0.80 points lower than those of non-attendees (B = −0.80, 95% CI −1.04 to −0.56, *p* < 0.001); in parallel, their odds of screening positive were 42.0% lower (OR = 0.58, 95% CI 0.46–0.73). In the post-partum period, however, the strong attendance by period interaction (β = 0.82, *p* < 0.001) essentially erased this benefit, yielding a derived post-partum OR of 0.98, with virtually no difference between groups. Figure 2 displays the predicted mean GAD-7 and EPDS scores for these two periods by attendance status.

A similar pregnancy-specific pattern emerged for depressive symptoms. Class attendance was linked to a 1-point reduction in EPDS scores (B = −1.00, 95% CI −1.30 to −0.70, *p* < 0.001) and a 55.0% decrease in the odds of screening positive (OR = 0.45, 95% CI 0.35–0.57). Yet the interaction term (β = +1.19, *p* < 0.001) nullified this advantage after birth, producing a post-partum OR of 1.08.

### Symptom-profile outcome

**Figure 3:**
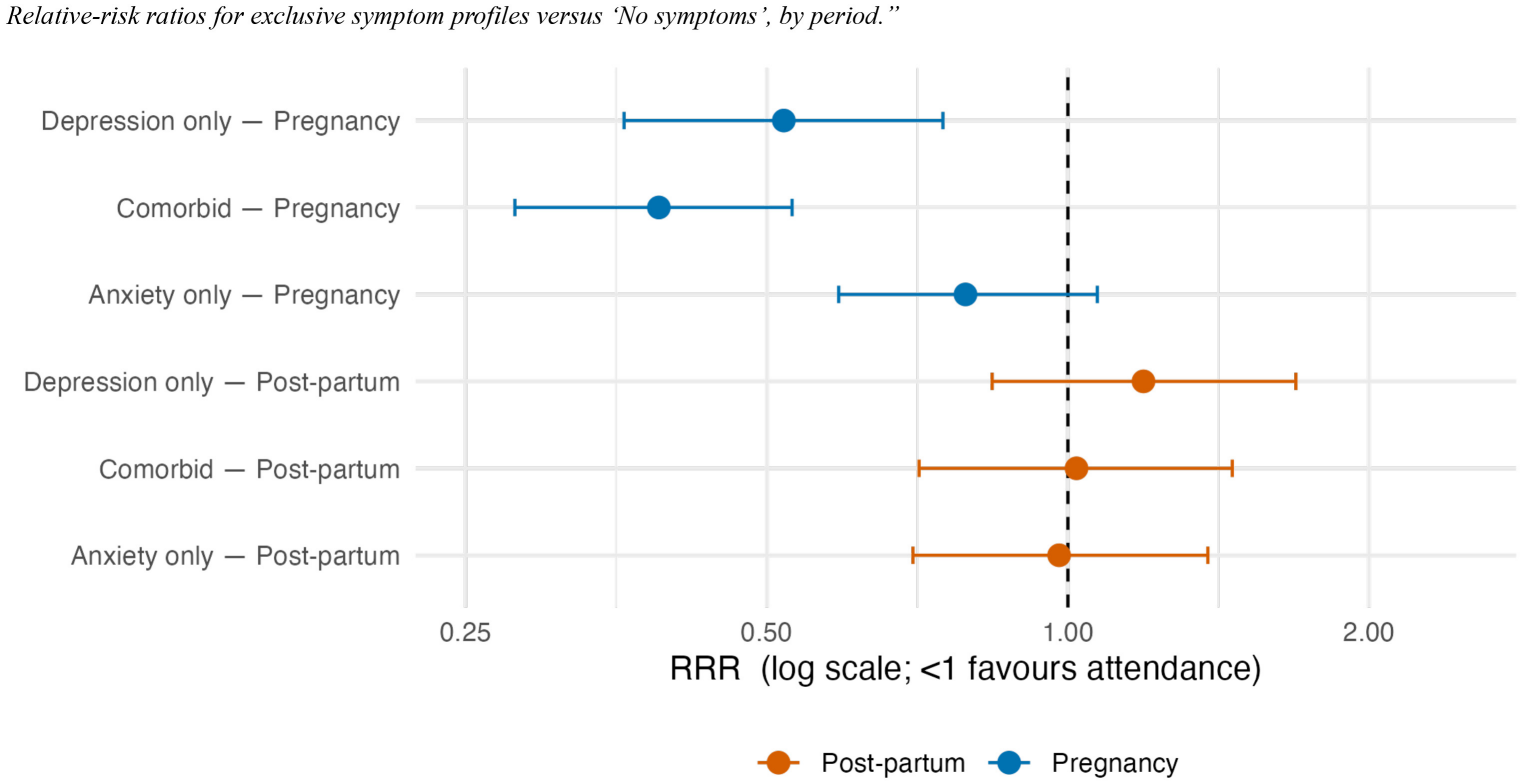
Relative-risk ratios for exclusive symptom profiles vs. “No symptoms,” by period.

Relative-risk ratios (vs. No symptoms) showed lower risks for depression-only (RRR = 0.52, 95% CI 0.36–0.75) and comorbid depression and anxiety (RRR = 0.39, 0.28–0.53), but no clear change in the anxiety-only profile (RRR = 0.79, 0.59–1.07). None of these differences was observed in the post-partum sample. Figure 3 visualizes these profile-specific RRRs and their 95% CIs.

### Absolute risk reduction

**Figure 4:**
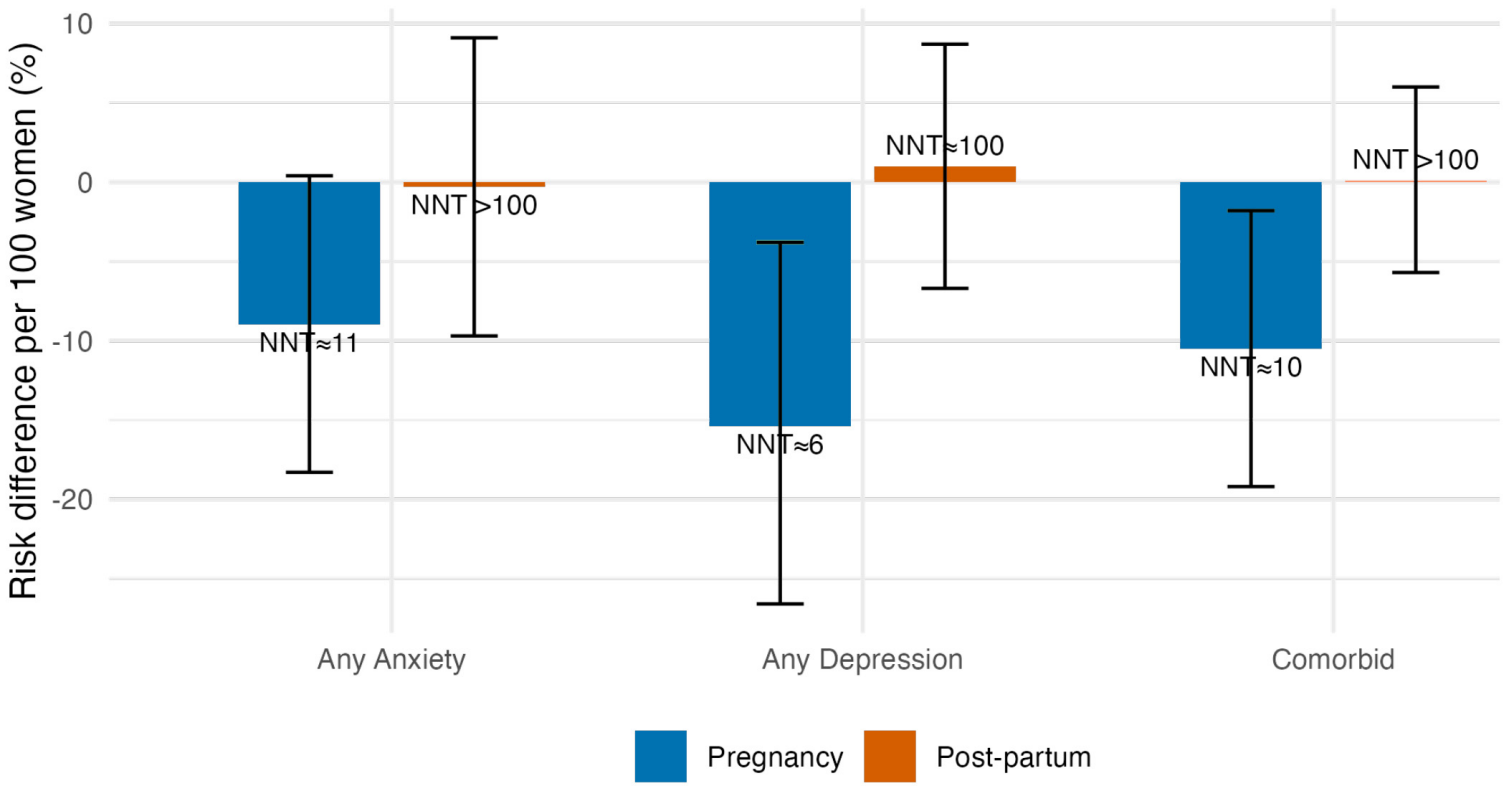
Risk differences per 100 women (bars ± 95% CIs) and implied number-needed-to-treat. Negative bars = risk reduction.

During pregnancy, attendance was associated with 9 fewer risk of anxiety cases (RD = −0.09, 95% CI −0.183 to +0.004; NNT ≈ 11) and 15 fewer risk of depression cases (RD = −0.154, −0.27 to −0.04; NNT ≈ 7) per 100 women. Post-partum risk differences hovered around zero, yielding NNTs > 100. This means that even under best-case assumptions, only one extra anxiety case would be prevented for roughly every 11 pregnant attendees, and benefits vanish in post-partum. These absolute differences and NNTs are illustrated in Figure 4.

### Propensity-score weighted (IPTW) analyses

**Figure 5:**
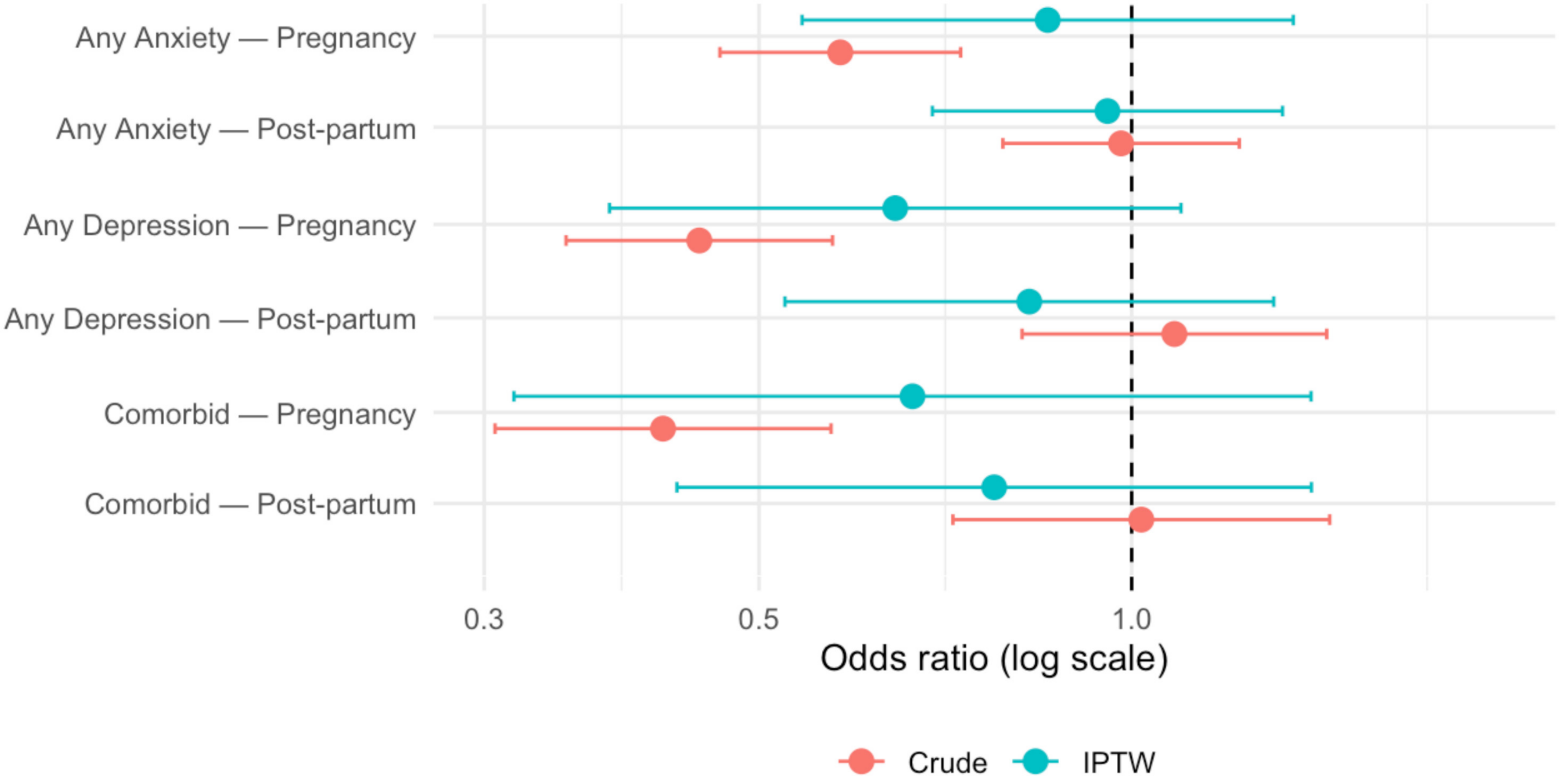
Crude and IPTW odds-ratios for antenatal-class attendance predicting anxiety, depression, and comorbidity, stratified by period. Dashed line = null effect.

Balancing on all covariates attenuated the pregnancy associations to non-significance: for women who were still pregnant, the IPTW OR for anxiety was 0.86 (95% CI 0.54–1.35), while the OR for depression was 0.64 (0.38–1.10). Adding the interaction term to obtain post-partum estimates yielded ORs of 0.96 (0.46–1.97) for anxiety and 0.83 (0.34–1.99) for depression, confirming no benefit after delivery. In other words, after inverse-probability weighting balanced the observed covariates, the associations attenuated and became non-significant, indicating that the crude advantages largely reflected baseline differences between women who did and did not enroll. Figure 5 juxtaposes these IPTW odds-ratios with the corresponding crude estimates, illustrating the attenuation.

### Sensitivity to unmeasured confounding

E-values indicated that an unmeasured factor associated with both class attendance and outcome by a risk ratio of ≈2 could fully explain the residual pregnancy associations (anxiety RR = 0.76, E-value = 1.95; depression RR = 0.67, E-value = 2.35; comorbid RR = 0.65, E-value = 2.47). These E-values show the pregnancy effects are only moderately robust: an unmeasured factor that roughly doubles both class attendance and mental-health status could wipe them out.

### Exploratory moderation and mediation analyses

The following results should be read as hypothesis-generating.

Among pregnant women who felt under-supported, classes were linked to clearly lower anxiety (ΔGAD ≈ −0.80, 95% CI −1.30 to −0.30). When perceived support was average or high, the anxiety difference shrank to essentially zero. For depression, the class gap was small and imprecise at all perceived support levels. These results suggest that women who are short on support seem to experience the most anxiety relief.

Primigravidas recorded a −0.5-point EPDS difference (95% CI −0.90 to −0.10), but the gap vanished for mothers who already had children. No parity pattern appeared for anxiety.

Low, medium, and highly educated individuals displayed similar effects; at most there was a non-significant trend toward a slightly larger anxiety benefit in the highest-education band.

In brief, antenatal classes appear to soothe prenatal anxiety only for women who feel short on partner- or friend-support; those with average or high support gain nothing extra. Depressive symptoms show a small benefit confined to first-time mothers, while parity is irrelevant for anxiety. Educational level does not meaningfully alter any effect. All subgroup findings are exploratory and should be viewed as hypothesis-generating.

## Discussion

This study investigated the effects of antenatal education classes, as an adjunct to routine obstetric and gynecological care, on anxiety and depressive symptoms among pregnant and postpartum women. It examined a large cohort of Italian and Italian-speaking women by comparing symptom levels between women who attended antenatal classes and those who did not. Widely used and validated self-report scales measured anxiety and depressive symptoms. Results indicate that participation in antenatal class provides a statistically significant but clinically modest effect in reducing anxiety and depressive symptoms during pregnancy. However, these benefits diminish significantly when differential selection is accounted for through propensity weighting and do not persist into the postpartum period. Specifically, while antenatal classes might offer protection against complex depressive and, especially, anxiety states during pregnancy, their effectiveness does not extend postpartum. The cross-sectional design, distinct pregnancy/postpartum cohorts, and moderate E-values suggest that the observed associations are likely not causal. Although routine antenatal classes may provide short-term psychological benefits during pregnancy, they are unlikely to prevent postpartum anxiety and depressive disorders without additional interventions.

The modest reductions in prenatal anxiety and depressive symptoms among attendees detected in our study are consistent with prior studies suggesting antenatal educational interventions can mitigate pregnancy-specific anxiety (Madhavanprabhakaran et al., 2017; Stoll et al., 2018) and improve childbirth-related outcomes like self-efficacy and pain management (Demirci et al., 2023; Alizadeh-Dibazari et al., 2023). Furthermore, the limited and transient psychological effects is consistent with recent reviews (Athinaidou et al., 2024; Rahmawati et al., 2025). The observed reduction in anxiety during the antepartum period confirms previous findings on the protective role of antenatal education (Rahmawati et al., 2025), particularly for primiparous women facing greater uncertainty about pregnancy risks, medical complications, and childbirth (Lafontaine et al., 2024; Khajehei et al., 2020). However, our result indicating no postpartum benefit in terms of depressive symptom severity contrasts preliminary meta-analytic findings (*k* = 3; *N* = 367) suggesting antenatal education may reduce postpartum depression (Alizadeh-Dibazari et al., 2023). Our findings are consistent with psychoeducational approaches reporting reduced anxiety and enhanced self-efficacy in prenatal settings (Diotaiuti et al., 2022), although adjusted estimates in our study suggest attenuation over time and limited persistence postpartum.

Methodologically, applying inverse-probability-of-treatment weighting significantly attenuated initial associations, revealing that baseline socio-demographic and psychological differences substantially confound the relationship between antenatal education and psychological outcomes. Moderate E-values further suggest susceptibility to unmeasured confounding, reinforcing caution in interpreting observed psychological benefits as strictly causal. Thus, the attenuation observed after propensity-score weighting suggests selection bias may partly explain previously reported positive outcomes.

Our exploratory moderation analyses provided insights into subgroups potentially benefiting more from antenatal education. Specifically, women reporting low perceived social support showed clearer reductions in anxiety following class attendance. This aligns with strong evidence that perceived social support is critical in buffering anxiety during pregnancy (Cena et al., 2020; McCarthy et al., 2021). The minimal benefits for those with higher perceived social support suggest antenatal education may serve as a compensatory resource, especially beneficial where other supports are lacking. Given that antenatal education in Italy is predominantly delivered in group formats, therapeutic factors specific to group interventions (Yalom and Leszcz, 2020), such as universality (i.e., the recognition that others share similar experiences), may be key mechanisms of action. The moderation observed with depressive symptoms in primiparous women aligns with the understanding that first-time mothers often face heightened vulnerability (Athinaidou et al., 2024; Barimani et al., 2018). Antenatal classes may thus specifically target initial anxieties and depressive symptoms associated with transitioning into motherhood.

### Clinical implications

Our findings suggest antenatal education should not be considered a standalone preventive measure for postpartum anxiety and depression. Instead, antenatal classes should be integrated into broader psychosocial support frameworks that emphasize systematic, evidence-based screening/assessment (Cena et al., 2021d) and timely referral for mental health services throughout the perinatal period. Given the observed weakening of effects detected after adjustment for confounders and their moderate robustness to unmeasured biases, refinement of antenatal curricula is recommended. Practical next steps include enhancing and standardizing antenatal programs with evidence-based content, extending educational support into the postpartum period, and integrating antenatal education with postpartum care programs, particularly for those initially benefiting from antenatal classes. Lastly, our exploratory analyses further suggest prioritizing socially isolated and primigravid women.

### Limitations and strengths

This study's findings should be interpreted considering some limitations.

The observational design of the study restricts causal inference despite adjustment. Furthermore, the self-reported nature of measures with possible social desirability bias and timing sensitivity, incomplete measurement of adherence to the intervention, limited power for some secondary outcomes and for postpartum follow-up; restricted the generalizability of the study.

Additionally, information regarding program content and delivery formats (e.g., in-person vs. online) was unavailable, highlighting variability in antenatal education programs and reinforcing the need for standardized yet personalized approaches. Despite inverse-probability-of-treatment weighting adjustments, self-selection bias remains, further underscoring the necessity for longitudinal randomized controlled trials to validate findings unequivocally.

However, some strengths can be considered as the large sample size and the use of comprehensive analytical techniques beyond logistic regression, enabling a nuanced understanding of clinical effects.

## Conclusion

Antenatal education classes are modestly effective in reducing anxiety and depressive symptoms during pregnancy, particularly benefiting first-time mothers and women with limited perceived social support. The transient nature of these beneficial effects emphasizes the need for standardized, evidence-based interventions integrated into routine perinatal psychological monitoring to achieve and maintain maternal mental health benefits.

## Data Availability

Data that support the findings of this study are available from the corresponding author upon reasonable request.

## Group members of Perinatal Mental Health Network group

Franca Aceti, Osservatorio Multicentrico per la Depressione Perinatale, Servizio di psicopatologia perinatale, Sapienza Università di Roma, Policlinico Umberto I, Roma, Italy; Ilaria Adulti, Osservatorio Multicentrico per la Depressione Perinatale, Università di Tor Vergata, Roma, Italy; Pietro Bagolan, Ospedale Pediatrico Bambino Gesù, Unità Operativa di Psicologia Clinica e diagnosi prenatale, Roma, Italy; Gina Barbano, UOC Infanzia Adolescenza Famiglia e Consultori, Distretto Treviso nord-sede di Oderzo, Azienda ULSS 2 Marca trevigiana, Treviso, Italy; Antonello Bellomo, Osservatorio Multicentrico Depressione Perinatale, UOC Psichiatria, Università di Foggia, Foggia, Italy; Marina Cattaneo, Consultorio familiare di Treviglio, ASST Bergamo Ovest, Bergamo, Italy; Elda Cengia, UOC Ostetricia e Ginecologia Ospedale di Belluno Azienda ULSS 1, Belluno, Italy; Flavia Adalgisa Distefano, UOC di Ginecologia ed Ostetricia dell'Ospedale Cristo Re, Roma, Italy; Angela Fabiano, Divisione Materno Infantile dell'Azienda Ospedaliera di Rilievo Nazionale e di Alta Specializzazione (ARNAS) “Garibaldi”, Catania, Italy; Alice Fent, UOC Ostetricia e Ginecologia Ospedale di Belluno Azienda ULSS 1, Belluno, Italy; Nicoletta Giacchetti, Servizio di psicopatologia perinatale Sapienza Università di Roma, Policlinico Umberto I, Osservatorio Multicentrico per la Depressione Perinatale, Roma, Italy; Laura Giusti, Università degli Studi de L'Aquila, Dipartimento di Medicina Clinica, Sanità Pubblica, Scienze della Vita e dell'Ambiente - MeSVA, L'Aquila, Italy; Antonella Grillo, Divisione Materno Infantile - ASP Catania, Catania, Italy; Teresa Grimaldi, Ospedale Pediatrico Bambino Gesù, Unità Operativa di Psicologia Clinica e diagnosi prenatale, Roma, Italy; Loredana Messina, UOC Ostetricia e Ginecologia dell'Ospedale Buccheri La Ferla Fatebenefratelli, Palermo, Italy; Marianna Mazza, Osservatorio Multicentrico Depressione Perinatale, UOC Psichiatria, Policlinico A. Gemelli, Roma, Italy; Angelo Marcheggiani, Consultorio di Campobasso - Ambulatorio di Ostetricia e Ginecologia, Campobasso, Italy; Cinzia Niolu, Osservatorio Multicentrico per la depressione perinatale, Università di Tor Vergata, Roma, Italy; Giovanna Picciano, Consultorio di Campobasso - Ambulatorio di Ostetricia e Ginecologia, Campobasso, Italy; Maria Pistillo, UOC Ostetricia e Ginecologia, ASP Enna, Enna, Italy; Rossana Riolo, Ambulatorio genitori senza depressione, ALSS 8-Berica, Vicenza, Italy; Rita Roncone, Università degli Studi de L'Aquila, Dipartimento di Medicina Clinica, Sanità Pubblica, Scienze della Vita e dell'Ambiente - MeSVA, L'Aquila, Italy; Gabriele Sani, Osservatorio Multicentrico Depressione Perinatale, UOC Psichiatria, Policlinico A. Gemelli, Roma, Italy; Melania Severo, Osservatorio Multicentrico Depressione Perinatale, UOC Psichiatria, Università di Foggia, Foggia, Italy; Martina Smorti, Dipartimento di Patologia Chirurgica, Medica, Molecolare e dell'Area Critica, Università di Pisa, Pisa, Italy; Damiana Tomasello, Divisione Materno Infantile - ASP Catania, Catania, Italy.
