# Supplementary material for: Effects of antenatal education on maternal anxiety and depression in pregnancy and postpartum period in Italy: modest and transient symptom reductions
Source: Front Psychol. 2026 Feb 12;16:1724202. doi: 10.3389/fpsyg.2025.1724202 (PMC12936865; doi:10.3389/fpsyg.2025.1724202)
Supplement: Supplementary file 1 [file Table_1.docx]

**Supplementary Table 1**

*Pregnancy: Attended vs. Not Attended Antenatal Classes (N = 4,169)*

| Variable | Level | No (*n*=2355) | Yes (*n*=1814) | *p* |
| --- | --- | --- | --- | --- |
| Age, mean (SD) | — | 33.04 (5.63) | 33.06 (5.28) | .892 |
| Nationality | Italian | 2114 (89.7%) | 1744 (96.1%) | <.001 |
|  | Non‑Italian | 242 (10.3%) | 70 (3.9%) |  |
| Education level | Primary | 356 (15.1%) | 102 (5.6%) | <.001 |
|  | Secondary | 1009 (42.8%) | 680 (37.5%) |  |
|  | Tertiary | 992 (42.1%) | 1033 (56.9%) |  |
| Marital status | Single | 309 (13.1%) | 140 (7.7%) | <.001 |
|  | Married/cohabiting | 2046 (86.9%) | 1675 (92.3%) |  |
| Household composition | Living alone | 26 (1.1%) | 22 (1.2%) | <.001 |
|  | With partner | 2209 (93.8%) | 1755 (96.8%) |  |
|  | Other family | 118 (5.0%) | 36 (2.0%) |  |
| Economic status | Low | 243 (10.3%) | 141 (7.8%) | .019 |
|  | Medium | 1420 (60.3%) | 1140 (62.8%) |  |
|  | High | 690 (29.3%) | 532 (29.3%) |  |
| Employment status | Unemployed/unstable | 890 (37.8%) | 461 (25.4%) | <.001 |
|  | Student/other | 167 (7.1%) | 145 (8.0%) |  |
|  | Employed | 1298 (55.1%) | 1206 (66.5%) |  |
| History of abortion | No | 1568 (66.5%) | 1368 (75.4%) | <.001 |
|  | Yes | 787 (33.5%) | 446 (24.6%) |  |
| Other living children | No | 1003 (42.6%) | 1475 (81.3%) | <.001 |
|  | Yes | 1351 (57.4%) | 339 (18.7%) |  |
| Planned pregnancy | No | 817 (34.7%) | 431 (23.8%) | <.001 |
|  | Yes | 1537 (65.3%) | 1383 (76.2%) |  |
| Support from friends/relatives | None | 115 (4.9%) | 30 (1.7%) | <.001 |
|  | Low | 245 (10.4%) | 149 (8.2%) |  |
|  | Moderate | 735 (31.2%) | 553 (30.5%) |  |
|  | High | 1260 (53.5%) | 1081 (59.6%) |  |
| Partner support | None | 61 (2.6%) | 33 (1.8%) | .122 |
|  | Low | 137 (5.8%) | 122 (6.7%) |  |
|  | Moderate | 502 (21.3%) | 415 (22.9%) |  |
|  | High | 1653 (70.2%) | 1245 (68.6%) |  |
| Current psychotropic medication | No | 2254 (95.7%) | 1789 (98.6%) | <.001 |
|  | Yes | 101 (4.3%) | 25 (1.4%) |  |
| Past psychiatric diagnosis | No | 1990 (84.5%) | 1537 (84.7%) | .873 |
|  | Yes | 365 (15.5%) | 277 (15.3%) |  |
| Family history of diagnosis | No | 1935 (82.1%) | 1408 (77.6%) | <.001 |
|  | Yes | 421 (17.9%) | 406 (22.4%) |  |
