# Supplementary material for: Effects of antenatal education on maternal anxiety and depression in pregnancy and postpartum period in Italy: modest and transient symptom reductions
Source: Front Psychol. 2026 Feb 12;16:1724202. doi: 10.3389/fpsyg.2025.1724202 (PMC12936865; doi:10.3389/fpsyg.2025.1724202)
Supplement: Supplementary file 2 [file Table_2.docx]

**Supplementary Table 2**

*Postpartum: Attended vs. Not Attended Antenatal Classes (N = 5,520)*

| Variable | Level | No (*n*=2732) | Yes (*n*=2788) | *p* |
| --- | --- | --- | --- | --- |
| Age, mean (SD) | — | 33.21 (5.42) | 32.87 (4.89) | .015 |
| Nationality | Italian | 2355 (86.1%) | 2643 (94.8%) | <.001 |
|  | Non‑Italian | 380 (13.9%) | 145 (5.2%) |  |
| Education level | Primary | 404 (14.8%) | 153 (5.5%) | <.001 |
|  | Secondary | 1344 (49.2%) | 1120 (40.2%) |  |
|  | Tertiary | 984 (36.0%) | 1515 (54.3%) |  |
| Marital status | Single | 232 (8.5%) | 173 (6.2%) | .001 |
|  | Married/cohabiting | 2500 (91.5%) | 2615 (93.8%) |  |
| Household composition | Living alone | 30 (1.1%) | 14 (0.5%) | <.001 |
|  | With partner | 2640 (96.5%) | 2735 (98.2%) |  |
|  | Other family | 62 (2.4%) | 39 (1.3%) |  |
| Economic status | Low | 191 (7.0%) | 103 (3.7%) | <.001 |
|  | Medium | 1545 (56.5%) | 1477 (52.9%) |  |
|  | High | 996 (36.5%) | 1208 (43.4%) |  |
| Employment status | Unemployed/unstable | 889 (32.5%) | 421 (15.1%) | <.001 |
|  | Student/other | 137 (5.0%) | 159 (5.7%) |  |
|  | Employed | 1706 (62.5%) | 2208 (79.2%) |  |
| History of abortion | No | 1823 (66.7%) | 2151 (77.2%) | <.001 |
|  | Yes | 909 (33.3%) | 637 (22.8%) |  |
| Other living children | No | 716 (26.2%) | 2395 (85.8%) | <.001 |
|  | Yes | 2016 (73.8%) | 393 (14.2%) |  |
| Planned pregnancy | No | 774 (28.3%) | 508 (18.2%) | <.001 |
|  | Yes | 1958 (71.7%) | 2 280 (81.8%) |  |
| Support from friends/relatives | None | 77 (2.8%) | 50 (1.8%) | <.001 |
|  | Low | 314 (11.5%) | 223 (8.0%) |  |
|  | Moderate | 845 (30.9%) | 740 (26.5%) |  |
|  | High | 1496 (54.7%) | 1775 (63.7%) |  |
| Partner support | None | 27 (1.0%) | 20 (0.7%) | <.001 |
|  | Low | 126 (4.6%) | 84 (3.0%) |  |
|  | Moderate | 566 (20.7%) | 379 (13.6%) |  |
|  | High | 2013 (73.7%) | 2305 (82.7%) |  |
| Current psychotropic medication | No | 2673 (97.7%) | 2751 (98.6%) | .012 |
|  | Yes | 59 (2.3%) | 37 (1.4%) |  |
| Past psychiatric diagnosis | No | 2506 (91.7%) | 2520 (90.3%) | .067 |
|  | Yes | 226 (8.3%) | 268 (9.7%) |  |
| Family history of diagnosis | No | 2386 (87.3%) | 2339 (83.9%) | <.001 |
|  | Yes | 346 (12.7%) | 449 (16.1%) |  |
